# Multiple Mating and Family Structure of the Western Tent Caterpillar, *Malacosoma californicum pluviale*: Impact on Disease Resistance

**DOI:** 10.1371/journal.pone.0037472

**Published:** 2012-05-24

**Authors:** Michelle T. Franklin, Carol E. Ritland, Judith H. Myers, Jenny S. Cory

**Affiliations:** 1 Department of Biological Sciences, Simon Fraser University, Burnaby, British Columbia, Canada; 2 Department of Forest Sciences, Genetic Data Centre, University of British Columbia, Vancouver, British Columbia, Canada; 3 Department of Zoology, University of British Columbia, Vancouver, British Columbia, Canada; University of Lausanne, Switzerland

## Abstract

**Background:**

Levels of genetic diversity can strongly influence the dynamics and evolutionary changes of natural populations. Survival and disease resistance have been linked to levels of genetic diversity in eusocial insects, yet these relationships remain untested in gregarious insects where disease transmission can be high and selection for resistance is likely to be strong.

**Methodology/Principal Findings:**

Here we use 8 microsatellite loci to examine genetic variation in 12 families of western tent caterpillars, *Malacosoma californicum pluviale* from four different island populations to determine the relationship of genetic variability to survival and disease resistance. In addition these genetic markers were used to elucidate the population structure of western tent caterpillars. Multiple paternity was revealed by microsatellite markers, with the number of sires estimated to range from one to three per family (mean ± SE  = 1.92±0.23). Observed heterozygosity (H_O_) of families was not associated to the resistance of families to a nucleopolyhedrovirus (NPV) (r = 0.161, F_1,12_  = 0.271, *P* = 0.614), a major cause of mortality in high-density populations, but was positively associated with larval survival (r = 0.635, F_1,10_  = 5.412, *P* = 0.048). Genetic differentiation among the families was high (F_ST_ = 0.269, *P*<0.0001), and families from the same island were as differentiated as were families from other islands.

**Conclusion/Significance:**

We have been able to describe and characterize 8 microsatellite loci, which demonstrate patterns of variation within and between families of western tent caterpillars. We have discovered an association between larval survival and family-level heterozygosity that may be relevant to the population dynamics of this cyclic forest lepidopteran, and this will be the topic of future work.

## Introduction

For gregarious or colonial animals the opportunities for disease transmission among individuals are likely to be high and thus, selection for resistance to disease is also expected to increase [1], [2], although exceptions occur [3]. Resistance to disease could be genetically determined by specific genes, or by the levels of heterozygosity of individuals that might be associated with their overall vigour [4]–[7]. It has been proposed that increased genetic variability associated with polyandry reduces the risk of parasitism as selection imposed by parasites favours rare genotypes [8], [9]. This prediction, often discussed in the context of the Red Queen hypothesis, has been the stimulus for considerable research on disease resistance and polyandry in eusocial insects such as bees, ants, and termites [10]–[14]. Experiments manipulating colony heterogeneity in eusocial species have found genetic diversity to be an important determinant of disease resistance [13], [15]. In addition, studies of clonal animals have found negative relationships between genetic polymorphism and parasite infection [7], [16].

**Figure 1:**
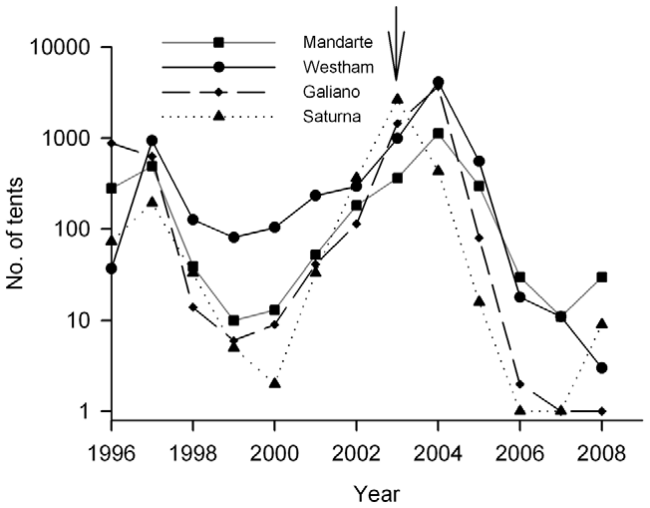
Population cycles of *Malacosoma californicum pluviale* tents. Number of *Malacosoma californicum pluviale* tents per study site on four islands in southwestern British Columbia, Canada from 1996 to 2008 [20]. The egg masses used to obtain larvae for the genetic analysis were produced by moths in 2002 and would have been part of the larval population in 2003 (arrow).

Tests of disease resistance in field populations of animals, particularly invertebrates, is challenging and data are limited, although detailed studies have been carried out on *Daphnia magna* [6], [17], [18] and the fresh water snail, *Potamopyrgus antipodarum* [16], and to a more limited extent, the damselfly, *Coenagrion puella* [19]. Recently we demonstrated significant among family variation in the resistance of western tent caterpillars, *Malacosoma californicum pluviale* to infection by a nucleopolyhedrovirus (NPV) that occurs regularly at high host population densities [20]. Western tent caterpillars fluctuate with a 6 to 11 year periodicity in southwestern British Columbia Canada [20], [21] (Figure 1). They are gregarious and females lay a single egg mass and larvae stay together during the larval stages to form a tent. We found that the resistance of families tended to increase following the viral epizootic that occurred in peak populations. This indicates that selection through virus mortality may select for more resistant individuals.

Given the extensive work on polyandry, genetic variation and potential disease resistance in colonial insects [10], [15] we wanted to determine if polyandry occurred in the gregarious western tent caterpillars and if this could increase the genetic variability within families. Opportunities for multiple mating in western tent caterpillars are likely to be constrained by the short life span of the adult moth and long copulation times. However, in the closely related eastern tent caterpillar (*M. americanum*) investigation of allozyme variation revealed that multiple mates were common [22]. In spite of this, sperm from one male dominated the fertilization of eggs of the female and thus genetic variation of families was not increased [22]. However, the low levels of polymorphism of allozyme markers may have been insufficient to show underlying patterns.

Genetic studies that evaluate hypotheses to explain population fluctuations in forest Lepidoptera remain underrepresented in the literature. Microsatellite markers allow for the evaluation of levels of neutral variation, with greater resolution than allozymes, and we have developed eight loci for western tent caterpillars to explore associations between genetic variation and fitness. We have used these microsatellite variants to determine (1) levels of genetic variability within and among families, (2) the relationship between genetic variability and the resistance of the families to nucleopolyhedroviral infection, (3) the relationship between genetic variability and the larval survival to the 4^th^ instar of development, (4) the extent of polyandry among families and (5) the association of genetic variability to the density of populations in the field. This study of the neutral genetic variation among families of tent caterpillars from different island populations can also be used to assess the levels of geographic subdivision that occur among families from the four islands surveyed.

Here we describe microsatellite variants that we have used to explore the potential relationships between genetic variability (observed levels of heterozygosity) and fitness characteristics of the western tent caterpillar. We test the hypothesis that increased heterozygosity is associated with greater resistance of families to nucleopolyhedroviral infection and to better survival. Contrary to our predictions, disease resistance was not related to genetic heterozygosity at the family level. Larval survival, however, was positively correlated with observed heterozygosity.

## Methods

### Ethics statement

Specific permits were not required for field collections of egg masses and permission was not required to obtain collections at all field sites. Because tent caterpillars are considered to be minor pests the public does not value them and removal of egg masses is in fact valued. They have neither endangered nor protected status. Collection sites are roadsides and “waste” areas.

**Figure 2:**
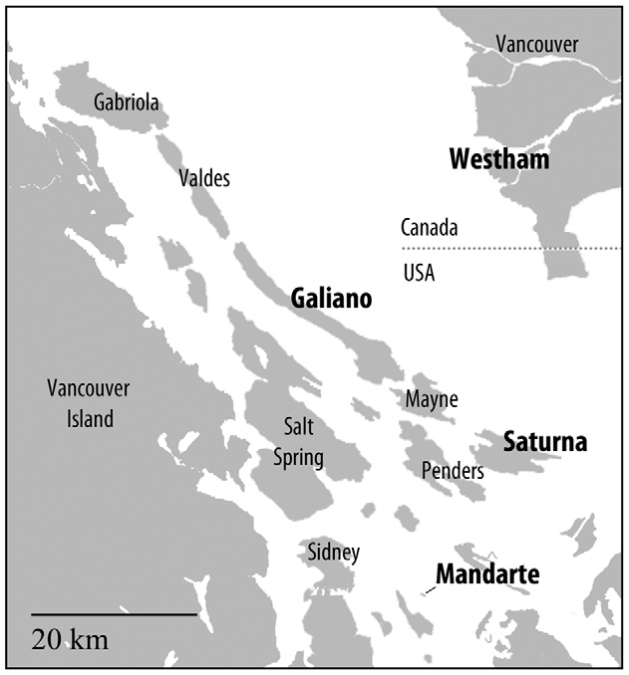
Map of the study area. *Malacosoma californicum pluviale* egg masses were collected from Galiano, Mandarte, Saturna, and Westham Island (bold font) along the west coast of British Columbia.

### Egg mass collection and rearing

Egg masses were collected from four islands in southern British Columbia, Canada during February 2003: Galiano Island (48.56N, 123.129W) from red alder, *Alnus rubra*, and wild cherry, *Prunus emarginata*, Saturna Island (48.778N, 123.129W) from red alder, Mandarte Island (48.634N, 123.288W) on the Haro Straight east of southern Vancouver Island from wild rose, *Rosa nutka*, and Westham Island (49.095N, 123.177W) in the delta of the Fraser River, south of Vancouver from crab apple, *Malus diversifolia* and hawthorn, *Crataegus monogyna* (Figure 2). These populations have been monitored annually by counting the number of tents in the specific sample areas or over the whole 7 ha for Mandarte Island (Figure 1) [20]. Egg masses were held at ambient temperature in a protected, outdoor location until April when they were brought into the laboratory for rearing. Egg masses were surface sterilized to remove external pathogens by placing them in a 0.8% bleach solution for 5 minutes. The families were reared separately in 1 L waxed paper cups and fed red alder *ad libitum.* The number of larvae that survived to the 4^th^ instar of development was counted and a sample of ten 4^th^ instar larvae were killed by freezing and stored individually at −20°C until DNA extraction and analysis. The number of eggs per mass was counted after the larvae had hatched. Survival of the family groups up to the time the sample was taken was determined from the total number of 4^th^ instar larvae in each family divided by the number of larvae that had hatched from the egg mass for that family.

The remaining 4^th^ instar larvae in each family were challenged with *M. c. pluviale* NPV (McplNPV) in bioassays that have been reported elsewhere [20]. For this, each larva was exposed to one of five doses of McplNPV (45 000, 22 500, 11 250, 5 625 or 1 407 virus occlusion bodies) or water control, applied in 3 μl to a 8 mm leaf disc cut from red alder leaves. Following consumption of the leaf disc and the virus dose, larvae were reared individually at room temperature with a natural light cycle, fed on alder leaves as necessary, and monitored for 15 days. The dose-response curves and the dose that killed 50 percent of the individuals in each family (LD_50_) were determined using generalized linear models with a binomial distribution and a logit link structure (JMP 6, SAS Institute). There was no interaction effect with dose, thus the dose-response lines were parallel, enabling LD_50_s to be compared across families and sites [20].

### Marker cloning

A repeat enriched library was constructed at the Genetic Identification Service (GIS, Chatsworth, CA USA) from a single caterpillar. Caterpillar DNA was isolated using DNeasy Blood and Tissue® kit (QIAGEN, Germantown, MD, USA). Digested DNA (350–700 bp fragments) was incorporated into *E. coli* strain DH5α (ElectroMax^TM^, Invitrogen) by electroporation. Colonies were enriched for four distinct oligonucleotide probes (CA, GA, ATG, TAGA). Ninety-nine colonies were sequenced and of these 83 contained microsatellites (GenBank Accession No. JN861644 – JN861726). Primers were designed for a subset of the microsatellites using DesignPCR version 1.03 (Research Genetics Inc.). Twenty-four microsatellites were tested for polymorphism using library DNA and seven additional individuals. Polymerase chain reactions (PCR) for all microsatellites used 2 ng of genomic DNA, 0.2 mM dNTP, (1X) PCR buffer, 0.025 units of Bio Taq, 2 mM MgCl_2_, 0.6 μmol each primer, (1X) sucrose/cresol red, and 5.15 μL of H_2_O in a total reaction volume of 10 μL. The PCR conditions were as follows: 94°C for 3 min, cycled 35 times at 94°C for 40 sec, 55 to 57°C for 40 sec, 72°C for 30 sec, with a final extension of 72°C for 4 min. Products were run on a 3% agarose gel and reviewed for polymorphism.

### DNA extraction and microsatellite analysis

DNA was extracted using DNeasy Blood and Tissue® kit (QIAGEN, Ontario, Canada) from 10 caterpillars from each of the 12 families. One individual from each of eight tent caterpillar families were used to further screen 21 of the microsatellites for polymorphism at the Genetic Data Centre (University of British Columbia, Vancouver, Canada). Thirteen of the 21 microsatellites had low polymorphism (2 to 4 alleles per locus) and were not investigated further. The other eight microsatellites showed a high level of polymorphism and therefore were selected for the investigation of the 12 tent caterpillar families, which were arbitrarily selected from a total of 30 families (see Table 1 for GenBank Accession No.). PCR reactions for all microsatellites used 10 ng of genomic DNA, 0.0625 mM dNTP, (1X) PCR Reaction buffer (Bio Basic Inc. ON, Canada), 0.25 units of Taq (Bio Basics Inc.), 0.75 or 1 mM MgCl_2_ (Fermentas, ON, Canada; optimized for each locus, see Table 2), 0.15 pmol of fluorescently labeled forward primer (IRDye® 700 or 800, Integrated DNA Technologies, CA, USA), 3 pmol reverse primer (Eurofins MGW Operon, Huntsville, AL, USA), 6.15 or 6.25 μL H_2_0 for a total reaction volume of 10 μL. PCR conditions were as follows: 95°C for 3 min, cycled 30–35 times at 94°C for 40 sec, 55 to 57°C for 40 sec, 72°C for 30 sec, with a final extension of 72°C for 4 min (optimized for each locus, see Table 2).

**Table 1:** Microsatellite Characteristics.

Primer name	Repeat motif	Primer sequence 5′–3′	Size range (bp)	*n_a_*	Freqs (range)	R_T_	H_E_	H_O_	F_IS_	GenBank no.
A106	CA_(25)_	**F**: ACTCGGTAAACCGTAAACTC	290–470	18	0.005–0.125	8.092	0.744	0.914	−0.228	JN861680
		R: TTCCACACTAGGGGTAAAG								
A113	GT_(13)_	**F**: CTATTTCCGCAAAGGAGTTG	221–237	7	0.017–0.478	4.416	0.521	0.684	−0.313	JN861683
		**R**: GTGCCGTTGTAGTGCTCTG								
A117	TA_(6)_CA_(11)_N_(19)_CA_(5)_gAC_(7)_	**F**: TGTCCTGGAGTGGAGACC	231–273	15	0.013–0.237	6.977	0.660	0.747	−0.131	JN861685
		R: ACTTGGATACATCGGTGTTG								
B6	CT_(6)_N_(12)_CT_(12)_	**F**: CGGAGACGAGATTTGTATGAG	267–277	7	0.004–0.411	4.521	0.508	0.511	−0.006	JN861654
		**R**: CGTTGCTTAATATCGGTGTTC								
B101	(GA)_20_	**F**: TTTTACCGAGGATTTCATTGTG	143–181	11	0.004–0.307	5.239	0.580	0.677	−0.167	JN861695
		**R**: CGGCTATTCAAATACTCCCC								
B103	CT_(13)_N_(18)_TA_4_tg(TA)_4_	**F**: GGCTCGTATTTTCTTTCGTTC	180–196	7	0.013–0.521	4.414	0.558	0.631	−0.131	JN861697
		**R**: GCCTATGTGGTGTATGTCAGC								
B108	(CT)_14_	**F**: GGCGGAGCTACTATTGAAAATC	174–186	6	0.017–0.385	3.996	0.530	0.393	0.259	JN861701
		**R**: CTGGAAACCGTTTATGAAACAG								
B121	(AG)_9_	**F**: TTGAGCGTTTGGAGTAACTG	275–281	4	0.057–0.726	3.023	0.351	0.244	0.307	JN861708
		**R**: ATAAAGAGGGCGTGAGATG								

Summary of microsatellite characteristics for *Malacosoma californicum pluviale.*

F: forward primer; R: reverse primer; size range of observed alleles; n_a_, number of observed alleles and their frequency range; R_T_, rarefied allelic richness; H_E_, expected heterozygosity, H_O_, observed heterozygosity, GenBank accession number.

**Table 2:** Polymerase chain reaction conditions for microsatellites.

Primer name	[MgCl_2_] mM	No. of PCR Cycles	T_a_ (°C)
A106	0.75	30	56
A113	0.75	33	57
A117	0.75	33	57
B6	1.00	33	57
B101	1.00	30	57
B103	1.00	30	57
B108	1.00	33	57
B121	1.00	35	58

Summary of polymerase chain reaction amplification (PCR) conditions for the eight microsatellite loci used to examine 12 *Malacosoma californicum pluviale* families.

[MgCl_2_], magnesium chloride concentration; Ta, annealing temperature.

Amplified products were denatured and run on a 6% polyacrylamide gel electrophoresis for 1.5 hours on a LI-COR 4300 automated sequencer (LICOR Inc., NE, USA) with a minimum of four size standards (50–350 bp or 50–700 bp LICOR Inc.) per 64 well gel and two to four reference samples. Gels were scored using SAGA 2.0 for microsatellites (LICOR). The accuracy of marker information was verified by repeating PCR and retyping samples within families for markers where multiple paternity was inferred (∼20% of samples verified).

### Sequencing

Sequences were obtained from four individuals at locus A106 to determine the cause of the large range of allele sizes (290–470 bp). Two of the individuals (Mandarte family 4: M4.4, M4.5) sequenced were homozygous for alleles 310 bp in size and the other two (Mandarte family 6: M6.2, M6.3) were homozygous for alleles 470 bp in size. PCR reactions used 10 ng of genomic DNA, 0.0625 mM dNTP, (1X) PCR buffer (Bio Basic Inc. ON, Canada), 0.25 units of Taq (Bio Basic Inc.), 2 mM MgCl_2_, 3 pmol forward and reverse primers (Eurofins MGW Operon, AL, USA), 6.25 μL H_2_0 for a total reaction volume of 10 μL. PCR conditions were as follows: 95°C for 3 min, cycled 35 times at 94°C for 40 sec, 56°C for 40 sec, 72°C for 30 sec, with a final extension of 72°C for 4 min. Four, 10 μL reactions were performed for each individual sequenced and amplified products were pooled prior to purification with a sodium acetate precipitation, and sent to Eurofins MWG Operon (AL, USA) for sequencing. Sequences were aligned using Bioedit Sequence Alignment Editor [23], a BLASTn search was performed to find significantly similar sequences and sequences were deposited in the GenBank database (GenBank Accession No. JN861727 – JN861730).

### Summary genetic data

To test for a step-wise mutation model the frequency distribution for each locus was constructed and normality was assessed visually and tested statistically using the Shapiro-Wilk normality test [24] in JMP version 8.0.2 (SAS Institute Inc. 2009). Genepop 4.0.10 [25], [26] was used to test for linkage disequilibrium among locus pairs within populations and across all populations and significance was assessed using Fisher's exact test [25].

GenoDive version 2.0b20 [27] was used to estimate the following genetic diversity measures: number of observed alleles (N_a_), number of effective alleles (N_e_), allele frequency range, expected heterozygosity (H_E_) [28], and observed heterozygosity (H_O_) for each locus and family group. Mean rarefied allelic richness (R_T_) was calculated for each loci and family in FSTAT 2.9.3.2 [29]. Several of these measures were significantly intercorrelated (Table S1) and therefore we chose to use observed heterozygosity, which is most often used to examine the relation between patterns of genetic variability and fitness characteristics. We tested for correlations between observed heterozygosity and population size in 2002, LD_50_ for NPV exposure, and survival in JMP 8.0.2. We used population size estimates from 2002, since the egg masses collected were the result of matings in 2002. Population size and LD_50_ were log transformed and survival was arcsine square root transformed to ensure that the assumption of normality was met. We performed post-hoc power analyses in SAS version 9.3 (SAS Institute Inc. 2010) to determine the statistical power for each correlation examined and determined the sample size that would have been required to achieve 80% power.

Global and pairwise F_ST_ values were estimated in Arlequin version 3.11 [30] for each family group, excluding loci with greater than 5% missing data. Significant differences between pairwise F_ST_ values were tested by performing 10 000 random permutations and the Bonferroni correction was used to account for multiple comparisons. Analysis of molecular variance (AMOVA) was performed in Arlequin version 3.11 to test for hierarchical genetic structure among families from the four different islands and significance was assessed by performing 10 000 random permutations of the data.

The inbreeding coefficient *f*, also referred to as F_IS_, was used to test for Hardy-Weinberg equilibrium (HWE) for each family-locus combination and overall for families and loci [31]. Significance at the 5% level was assessed by performing 96 000 random permutations of alleles among samples and the Bonferroni correction was applied.

### Paternity analysis

The minimum number of fathers was reconstructed for each family with unknown parental genotypes using an exhaustive search algorithm based on Mendelian segregation and population genotypic frequencies in GERUD 2.0 [32]. As recommended in the user manual, the initial parental reconstruction was performed using the three most polymorphic loci. Several combinations of loci were used when more than three loci had similar levels of polymorphism. GERUD 2.0 does not handle missing data and thus individuals with missing genotypes or loci for which data were missing for three or more individuals were excluded from the analyses. Correlation analysis was used to explore the relationships between the number of sires estimated and allelic richness (R_T_), disease resistance, and population size in 2002 (log transformed) (JMP 8.0.2). Allelic richness was used instead of observed heterozygosity because it is more sensitive to changes in the number of alleles. Post-hoc power analyses were performed in SAS 9.3 to determine the statistical power of each correlation and the sample size required to achieve 80% power.

## Results

### Microsatellite characterization

Microsatellites were identified in 83 of the 99 cloned colonies and contained di, tri, and tetra nucleotide repeats. Primers could not be designed for 23 of the microsatellites due to sequence duplication and unsuitable flanking regions for primer design. Twenty-one microsatellites were tested for polymorphism and of those, eight di-nucleotide loci were selected based on polymorphism for further analysis. BLASTn results produced no significant matches to coding regions for these eight microsatellites.

A step-wise mutation model may be inferred for microsatellite markers if their size frequency distribution is normally distributed. The Shapiro-Wilk normality test indicated that one of the eight loci (A106) we examined was not normally distributed (W = 0.625, *P*<0.0001) and rather shows a bi-modal distribution.

Alleles ranged in size from 143 to 470 bp, with alleles from locus A106 exhibiting an extremely large size range (290–470 bp) (Table 1). A comparison of sequences from locus A106 identified a large insertion of approximately 270 bp in individuals harbouring the large sized allele (470 bp) when sequences were aligned with those individuals where the small sized allele (310 bp) was present. A BLASTn search of the insertion produced no significant matches to coding sequence.

The number of observed alleles (N_a_) ranged from 4 to 18 and rarefied allelic richness (R_T_) varied from 3.0 to 8.1 for each locus (Table 1). Expected (H_E_) and observed heterozygosity (H_O_) ranged from 0.35 to 0.74 and 0.24 to 0.91, respectively. Observed heterozygosity was higher than the expected heterozygosity for all but two (B108 and B121) of the eight loci examined. None of the loci, however, showed significant departures from Hardy-Weinberg equilibrium (F_IS_  = −0.314 to 0.295; *P*>0.05). In addition, there was no evidence of linkage disequilibrium between any pairs of loci (*P*>0.05).

### Paternity analysis

**Figure 3:**
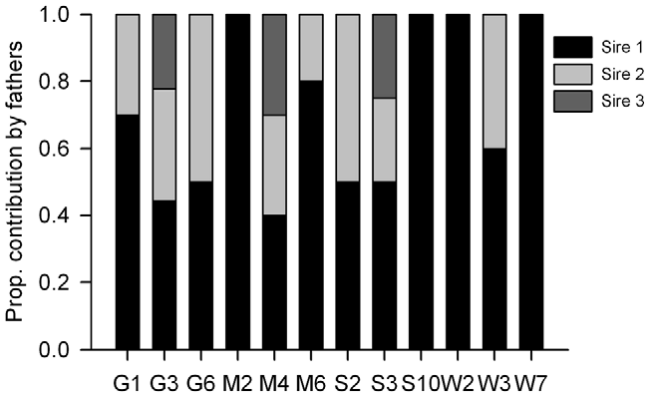
Paternity of offspring from collected egg masses. Proportional contribution of *Malacosoma californicum pluviale* sires to a sample of 10 progeny from egg masses collected from Galiano (G), Mandarte (M), Saturna (S), and Westham Island (W).

Multiple paternity was revealed by microsatellite analysis in eight of the 12 families studied (Figure 3). The minimum number of sires contributing to a family ranged from 1 to 3 (mean ± SE  = 1.92±0.23). Families from Westham Island had the lowest minimum number of fathers (mean ± SE  = 1.33±0.33), while families from Galiano Island had the highest (mean ± SE  =  2.33±0.33). In cases of multiple fathers, on average one sired approximately 60% of offspring and the other 40% for two fathers and for three fathers the average contribution of each sire was 45%, 29% and 26%, respectively.

Families with multiple sires were expected to have greater genetic diversity than those with a single sire. In agreement, the number of sires per family was positively correlated with allelic richness (r = 0.605, F_1,12_ = 5.79, *P* = 0.037, Power = 0.71). We also predicted that multiple paternity would be more prevalent in families at sites with the highest population size. However, there was no relation between the minimum number of sires and population size in 2002 (r = −0.307, F_1,12_ = 1.04, *P* = 0.331, Power  = 0.26). In addition, the association between the number of sires and disease resistance was weakly, but non-significantly positive (r = 0.295, F_1,12_ = 0.952, *P* = 0.352, Power  = 0.24). The statistical power of the correlations between the number of sires and population size in 2002 and disease resistance were relatively low and would require at least an additional 60 samples to potentially generate a significant result. However, it is difficult to obtain large data sets for gregarious insects because only one value can be used per family and therefore the required sample size represents the number of families and not individuals.

### Genetic diversity, population size, family structure and fitness correlates

Genetic diversity measures for 12 families from four island locations are described in Table 3. The mean number of effective alleles (n_e_) and allelic richness (R_T_) were greatest for families from Saturna Island, which had the highest population density in 2002 when egg masses were collected and reached peak density in 2003; a year prior to populations from other islands. The mean effective number of alleles and allelic richness were lowest on Mandarte Island, where the population is more isolated and reached peak size in 2004 (Table 3; Figure 1). The expected and observed heterozygosity followed a similar pattern and were greatest on average on Saturna and Galiano Islands and lowest on Mandarte Island. Within families, expected and observed heterozygosity levels were similar and there was no evidence of significant departures from Hardy-Weinberg equilibrium (F_IS_ = −0.230 to 0.120, *P*>0.05).

**Table 3:** Family genetic diversity estimates.

Island	Family	n_a_	n_e_	R_T_	H_O_	H_E_
Galiano	1	3.38	2.70	3.20	0.60	0.62
	3	3.50	2.69	3.38	0.54	0.60
	6	2.75	2.55	2.74	0.74	0.63
	Mean	3.21	2.65	3.11	0.63	0.62
Mandarte	2	2.75	2.23	2.71	0.65	0.53
	4	3.00	2.36	2.89	0.50	0.57
	6	2.25	1.99	2.24	0.46	0.44
	Mean	2.67	2.19	2.61	0.54	0.51
Saturna	2	4.13	3.02	3.76	0.73	0.68
	3	4.00	2.90	3.63	0.64	0.59
	10	2.50	2.06	2.46	0.53	0.46
	Mean	3.54	2.66	3.28	0.63	0.58
Westham	2	2.63	2.04	2.49	0.48	0.40
	3	3.00	2.51	2.87	0.61	0.58
	7	2.63	2.47	2.62	0.72	0.60
	Mean	2.75	2.34	2.66	0.60	0.53

Genetic diversity estimates for 12 *Malacosoma californicum pluviale* families based on 10 individuals sampled per family collected from four island locations in British Columbia, Canada.

n_a_, mean number of alleles; n_e_, mean number of effective alleles; R_T_, mean rarefied allelic richness, H_O_, mean observed heterozygosity; H_E_, mean expected heterozygosity.

We predicted that the high density of tent caterpillars in 2002, prior to the peak in population size would be positively associated with observed heterozygosity due to the increased availability of mates, however no relationship was identified (2002 H_O_: r = 0.067, F_1,12_  = 0.045, *P* = 0.835). Statistical power, however, was low (0.07) for this test and would have required in excess of 1000 families sampled to potentially generate a significant result. Within families, observed heterozygosity levels were similar and there was no evidence of significant departures from Hardy-Weinberg equilibrium (F_IS_ = −0.230 to 0.120, *P*>0.05).

**Figure 4:**
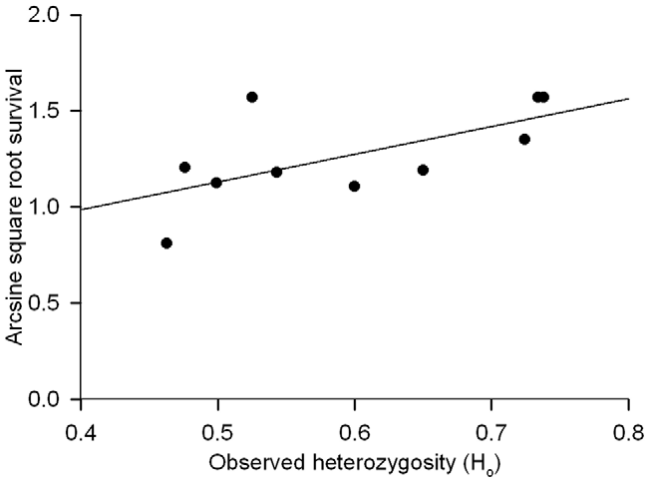
Observed heterozygosity and larval survival. Positive relationship between observed heterozygosity (H_O_) and survival from egg hatch to the 4^th^ instar of development for *Malacosoma californicum pluviale* families collected from monitoring sites on the islands of Galiano, Mandarte, Saturna, and Westham.

We expected higher genetic diversity to be related to greater resistance of families to viral infection, but found no relationship between the LD_50_s of each family and H_O_ (r = −0.162, F_1,12_  = 0.271, *P* = 0.614). The power of this test was low (0.128) and would have required samples from at least 230 more families. We also predicted that larval survival would be positively correlated with measures of genetic diversity. In agreement with this, we observed a positive multi-locus correlation for families between mean observed heterozygosity and survival from egg hatch to the 4^th^ instar of development (r = 0.635, F_1,10_ = 5.412, *P* = 0.048, Power  = 0.68; Figure 4).

The global F_ST_ estimate over all 12 families from the four islands indicated significant population differentiation among families (F_ST_ = 0.269, *P*<0.00001). With the exception of two families from Galiano Island (Gal 1 and Gal 3), all pairwise comparisons tested showed significant differentiation between families (Table 4; *P*<0.05). High levels of differentiation were often observed between families from the same island (for example comparing W2 and W3 F_ST_ = 0.420; comparing M4 and M6 F_ST_ = 0.351). Consistent with these results, differentiation among families from the same island contributed significantly (26%, *P*<0.00001). In addition, differences among families from different islands accounted for the greatest amount of variation (73%, *P*<0.00001) and there was no significant differentiation among islands (1%, *P* = 0.231).

**Table 4:** Family pairwise F_ST_ comparisons.

Family	G1	G3	G6	M2	M4	M6	S2	S3	S10	W2	W3	W7
**G1**	-											
**G3**	0.028*	-										
**G6**	0.160*	0.211*	-									
**M2**	0.267*	0.220*	0.296*	-								
**M4**	0.208*	0.185*	0.294*	0.248*	-							
**M6**	0.333*	0.347*	0.330*	0.321*	0.351*	-						
**S2**	0.200*	0.242*	0.234*	0.281*	0.249*	0.313*	-					
**S3**	0.149*	0.197*	0.202*	0.271*	0.214*	0.258*	0.236*	-				
**S10**	0.271*	0.280*	0.260*	0.353*	0.327*	0.233*	0.313*	0.258*	-			
**W2**	0.311*	0.323*	0.354*	0.317*	0.295*	0.394*	0.329*	0.292*	0.395*	-		
**W3**	0.159*	0.218*	0.206*	0.282*	0.297*	0.310*	0.265*	0.224*	0.310*	0.420*	-	
**W7**	0.152*	0.118*	0.251*	0.200*	0.127*	0.321*	0.240*	0.216*	0.321*	0.261*	0.236*	-

Pairwise F_ST_ values between *Malacosoma californicum pluviale* families collected from Galiano (G), Mandarte (M), Saturna (S), and Westham Island (W) on the west coast of British Columbia, Canada.

10 000 random permutations were used to test for significant differentiation among families.

* Populations are genetically differentiated at an overall significance level of *P*<0.05. Bonferroni correction was used to adjust for multiple comparisons.

## Discussion

Using eight new microsatellites, developed specifically for *M. c. pluviale*, we found that multiple paternity was common among families from four island populations. However, we found no relationship between viral resistance, as measured by the LD_50_, and the average observed heterozygosity of families. The average heterozygosity of families was, however, positively associated with larval survival. This finding is in agreement with other studies that have identified a positive association between genetic diversity and fitness benefits in eusocial insect species [13]–[15], but has not previously been studied in subsocial insects that lack complex social structure.

*Malacosoma c. pluviale* is primitively social and thus, individuals in families work together to build tents and mark food trails with chemical cues to attract siblings. But tent caterpillars lack complex social interactions such as grooming, food storage and provisioning behaviours that are characteristic of eusocial species [33]. Thus, if complex social behaviours are required to reduce infection, it is not surprising that we observed no relation between diversity and disease resistance in tent caterpillar families. However, if diversity influences individual immune physiology (or other disease resistance mechanisms), we may have failed to detect such a pattern, since our analysis was performed at the family level rather than the individual level. This was necessary as infection by NPV is lethal and thus resistance is evaluated as the lethal dose that kills 50% of the individuals. The weak, but non-significant, positive relationship between the LD_50_ and number of fathers represented in a family was largely determined by two families with particularly high resistance, one with two fathers and one with three.

Polyandry is prevalent in many lepidopteran species [34]. Previous investigation of the field-collected eastern tent caterpillars, *Malacosoma americanum,* identified, using allozyme variation, that at least 19% of females were multiply mated [22]. Low sperm penetrance, however, as inferred from the low number of effective mates observed per female, resulted in only a small effect on genetic structure [22]. In our study, the minimum number of *M. c. pluviale* sires per family ranged from one to three, with an average of two fathers per family. In contrast to the results for *M. americanum* [22], we found that all *M. c. pluviale* sires made a substantial genetic contribution to the offspring of egg clutches from multiply mated females. Our results are in line with those observed for other invertebrate species with internal fertilization [35].

The benefits of polyandry must outweigh the costs for it to be maintained [36]. The diversified bet hedging model predicts that multiply mated females that produce diverse offspring will achieve higher fitness than singly mated females when environments fluctuate [37]. This model is suitable for *M. c. pluviale* since its populations fluctuate and are subject to environmental perturbations such as changes in temperature, weather, and food availability [21], [38], [39]. Increased genetic variability could make them superior in activities that contribute to the success of the family. However, an experiment where the variability of eastern tent caterpillars families was increased by mixing individuals among families did not support this hypothesis [40]; family size influenced success but not variability among individuals.

A large body of research has focused on the benefits of multiple mating in relation to disease resistance in eusocial insects [10], [12]–[15], [41], where disease can spread rapidly due to colony living. The genetic variability vs. parasitism risk hypothesis predicts a fitness benefit of polyandry that is associated with increased genetic diversity because selection imposed by parasites will favour rare host genotypes [9]. In *Bombus terrestris* colonies where worker heterogeneity was manipulated by artificially inseminating queens, increased worker heterozygosity was associated with reduced infection to the common parasite, *Crithidia bombi* (Trypanosomatidae) [15]. Also in line with the genetic variability vs. parasitism risk hypothesis, inbred termite groups showed increased susceptibility to high concentrations of fungal conidia and had higher cuticular microbial loads when compared to outbred groups. The authors accredit this to better grooming by nestmates [13]. Not all studies of eusocial insects support this hypothesis however [11]. Although in tent caterpillar families individuals may vary in terms of activity and foraging in ways that would influence their vigour [42] and possibly their acquisition of pathogens, this variation is not apparently genetically based [33] and grooming and other hygienic activities do not occur. However, there is evidence that cadaver avoidance behaviour (of NPV-infected caterpillars) has a heritable component in other forest Lepidoptera [43] and it could be that behaviours more specifically related to reducing the risk of infection require more detailed investigation, particularly in gregarious caterpillars where the risk of infection is perceived to be higher.

Highly variable codominant microsatellite markers are well suited to this type of study of paternity and population structure [44], [45]. All of the microsatellites that we characterized for the western tent caterpillar were highly polymorphic (4 to 18 alleles per locus). With the exception of A106, which had a large insertion of 270 bp, all markers conformed to a step-wise mutation model and could be useful in future phylogenetic studies that assume this parameter. No associations were found between microsatellite sequences and known coding regions, suggesting that these markers are selectively neutral, however physical linkage with genes under selection is still possible [46].

Multi-locus heterozygosity fitness correlations that describe the association between individual heterozygosity and fitness related traits have been well documented in natural populations [47]. Here, we observed a significant correlation between the average multi-locus heterozygosity of families and the fitness trait, larval survival, in the laboratory. Our data do not permit the examination of an individual heterozygosity-fitness correlation because our measure of survival was performed at the family level. However, this does provide valuable insight into the relationship between average heterozygosity of individuals in families and fitness. For neutral microsatellite markers to be associated with fitness measures requires either linkage disequilibrium with loci under selection (local effect hypothesis) or a general effect of homozygosity on loci under selection across the genome (general effect hypothesis) [48]. *Malacosoma c. pluviale* populations go through boom and bust dynamics. The population trough that occurred in 1999–2000 may have increased the likelihood of strong linkage disequilibria [49] or the levels of heterozygosity could have increased with relaxed selection during the period of population increase following the trough and increased fitness associated with heterozygote advantage. Further work is required to distinguish these interpretations.

An additional finding of this study is that the greatest amount of genetic variation we observed was among families and this variation among families was similar within and among different islands. Genetic differentiation among populations of other cyclic forest Lepidoptera has been investigated using electrophoretic methods. For larch budmoth, *Zeiraphera diniana* in western Europe, F_ST_ values were higher (F_ST_ = 0.065) between populations feeding on two different host tree species than were the values among populations on the same host species (F_ST_ = 0.026 and 0.002) [50]. Populations of these two host races fluctuate in close synchrony much like the western tent caterpillars [51]. Eight populations of the pine beauty moth, *Panolis flammea* in England and Scotland were surveyed using four electrophoretic loci and significant variation existed among populations (F_ST_ = 0.109). The genetic similarity among populations of western tent caterpillars from different islands contrasts with these studies. Sample sizes in our study are small and only one year at high density has been studied.

The microsatellite variation described here can be used in future studies to determine if genetic variation and/or the number of mates varies with population density and if island populations are more differentiated at low population densities. The level of polyandry found here is surprising and could be under positive selection or could be the result of very high density and forced copulations. More behavioural work is required. Finally, it will be interesting to determine if these genetic markers can be used for other species of tent caterpillar such as the eastern tent caterpillar or the forest tent caterpillar, *M. disstria,* which periodically demonstrates population outbreaks across North America [52]. Genetic studies have been generally lacking in evaluations of hypotheses proposed to explain the population fluctuations of forest Lepidoptera.

## Supporting Information

Table S1 Intercorrelations between genetic diversity measures. Intercorrelations between genetic diversity measures for the 12 families of *Malacosoma californicum pluviale* families collected from Galiano, Mandarte, Saturna, and Westham Island on the west coast of British Columbia, Canada.
(PDF)
