# Supplementary material for: Multiple Mating and Family Structure of the Western Tent Caterpillar, Malacosoma californicum pluviale: Impact on Disease Resistance
Source: PLoS One. 2012 May 24;7(5):e37472. doi: 10.1371/journal.pone.0037472 (PMC3360058; doi:10.1371/journal.pone.0037472)
Supplement: Table S1 — Intercorrelations between genetic diversity measures. Intercorrelations between genetic diversity measures for the 12 families of Malacosoma californicum pluviale families collected from Galiano, Mandarte, Saturna, and Westham Island on the west coast of British Columbia, Canada. (PDF) [file pone.0037472.s001.pdf]

Table S1 Intercorrelations between genetic diversity measures. Intercorrelations between genetic diversity measures for the 12 families of *Malacosoma californicum pluviale* families collected from Galiano, Mandarte, Saturna, and Westham Island on the west coast of British Columbia, Canada.

	H_o	H_E	R_T	N_a	N_e
**H_o**	-				
**H_E**	0.762*	-			
**R_T**	0.427	0.757*	-		
**N_a**	0.390	0.702	0.994*	-	
**N_e**	0.645	0.901*	0.939*	0.918*	-

H_O, mean observed heterozygosity; H_E, mean expected heterozygosity; R_T, mean rarefied allelic richness; n_a, mean number of alleles; n_e, mean number of effective alleles

* Measures show significant correlation at $P < 0.05$.
